# Efficacy of Liu-zi-jue in Patients with 2019 Novel Coronavirus Pneumonia (COVID-19): structured summary of a study protocol for a randomized controlled trial

**DOI:** 10.1186/s13063-020-04383-2

**Published:** 2020-05-23

**Authors:** Shuaipan Zhang, Zhizhen Lv, Qingguang Zhu, Wuquan Sun, Fei Yao, Lei Fang, Yanbin Cheng, Zhiwei Wu, Min Fang

**Affiliations:** 1 Tuina Department, Yue yang Hospital of Integrated Traditional Chinese and Western Medicine, Shanghai University of Traditional Chinese Medicine, Shanghai, 200437 China; 2 Institute of Tuina, Shanghai Institute of Traditional Chinese Medicine, Shanghai, 200437 China; 3 Shanghai University of Traditional Chinese Medicine, Shanghai, 201203 China

**Keywords:** COVID-19, Randomized controlled trial, protocol, Liu-zi-jue, Conventional therapy

## Abstract

**Objectives:**

Efficacy of conventional treatment plus the complementary therapy Liu-zi-jue (a mind-body exercise) to treat patients with mild COVID-19.

**Trial design:**

The study is a single-center 2 arm, randomized controlled trial with parallel-group design.

## Participants

The trial will recruit 186 hospital patients with mild and general symptoms of COVID-19 pneumonia from 19th February to 31st July 2020. Diagnosis will follow the criteria of the National Health Commission & State Administration of Traditional Chinese Medicine Diagnostic and Treatment Protocol for Novel Coronavirus Pneumonia (The 7th Trial Version). Trial Inclusion criteria: (1) No restrictions on gender and age; (2) Patients diagnosed with mild pneumonia (slight clinical symptoms, no pneumonia manifestations on imaging), general pneumonia patients (with fever, respiratory tract symptoms, etc., imaging showed pneumonia but no multiple organ damage); (3) Hospitalized patients; (4) Volunteer to join the trial and sign the "informed consent; (5) Agree not to perform other exercise activities. Trial Exclusion criteria: (1) patients with severe diseases such as cardiovascular, cerebrovascular, hematopoietic, digestive system or mental illness; (2 )pregnant and lactating women; (3) respiratory frequency> 30 times /min, showing respiratory failure; (4) complicated with other organ failure requires treatment by respiratory intensive care unit (ICU); (5) those who do not want to join the trial. Participants will be recruited from inpatients at the Department of Infectious Diseases at the hospital in Hubei Province.

## Intervention and comparator

Patients in the control group with mild pneumonia, will be treated according to the 7^th^ Trial Version)" and given symptomatic supportive treatment. The intervention group will be instructed in performing Liu-zi-jue exercise therapy twice a day until 3 months after discharge. Liu-zi-jue, a low-risk traditional Chinese exercise, is widely used for the prevention and treatment of respiratory diseases as a complementary therapy. Its movements are relatively simple and are mainly composed of 6 groups of exercise combined with breathing and stretching, which is suitable for home practicing. There is no need to rely on expensive equipment and Liu-zi-jue can be practiced individually or in groups without the limitation of sports experience and age.

## Main outcomes

The primary outcome includes the Length of stay, Modified Borg Dyspnea Score, Fatigue Scale-14 Score, Patient Health Questionnaire-9 Score, which will evaluate the patient's respiratory symptoms and physical and mental health. The outcomes will be assessed at five points including the baseline, before treatment on the sixth day of hospitalization, the discharge day, 1 month after discharge and 3 months after discharge.

## Randomization

Patients will be recruited and randomly assigned to the conventional treatment group and the intervention group - conventional treatment plus Liu-zi-jue group in a 1:1 ratio. Researchers not delivering the intervention will get a random sequence number which is automatically generated by a random number generator (IBM Corp., Armonk, NY, USA). These will be sequentially placed in an opaque envelope. The research coordinator will open these random allocation envelopes and assign participants accordingly.

## Blinding (masking)

Participants and Liu-zi-jue trainers are unable to be blinded to group assignment due to the nature of this physical intervention. To reduce the risk of bias, evaluators, data managers, and statisticians will be unaware of group assignments in the outcome evaluation process and data analysis.

## Numbers to be randomized (sample size)

A total of 186 patients will be randomize 93 participants into the intervention group and 93 into the conventional treatment group.

## Trial Status

This trial is still recruiting patients now. The first patient was recruited on February 19 and is scheduled to complete recruitment on May 30. The protocol is Version 2, March 26.

## Trial registration

This trial was registered retrospectively in the Chinese Clinical Trial Registry on 26 April 2020. The registration number is ChiCTR2000032367.

## Full protocol

We declare that the full protocol is attached as an additional file, accessible from the Trials website (Additional file 1). In the interest in expediting dissemination of this material, the familiar formatting has been eliminated; this Letter serves as a summary of the key elements of the full protocol. The study protocol has been reported in accordance with the Standard Protocol Items: Recommendations for Clinical Interventional Trials (SPIRIT) guidelines (Additional file 2).

## Supplementary information

**Additional file 1.** Full protocol.

**Additional file 2.** SPIRIT 2013 Checklist.

**Additional file 3.** Proof of Ethics.

**Additional file 4.** Funding proof.

## Data Availability

Not applicable

