# Supplementary material for: Efficacy of Liu-zi-jue in Patients with 2019 Novel Coronavirus Pneumonia (COVID-19): structured summary of a study protocol for a randomized controlled trial
Source: Trials. 2020 May 23;21:416. doi: 10.1186/s13063-020-04383-2 (PMC7245505; doi:10.1186/s13063-020-04383-2)
Supplement: Supplementary file 1 — Additional file 1. Full protocol. [file 13063_2020_4383_MOESM1_ESM.docx]

Title:

Efficacy of Liu-zi-jue in Patients with 2019 Novel Coronavirus Pneumonia (COVID-19): study protocol for a randomized controlled trial

Author's full name:

Shuaipan Zhang^1,2*^, Zhizhen Lv^1 ,2*^, Qingguang Zhu ^2#^, Wuquan Sun ^1^, Fei, Yao^3^, Lei Fang^3^, Yanbin Cheng ^2^, Zhiwei Wu^1,2^, Min Fang^1,2,3#^

Authors’ institutional addresses

1 Tuina Department, Yue yang Hospital of Integrated Traditional Chinese and Western Medicine, Shanghai University of Traditional Chinese Medicine, Shanghai 200437, China. 2 Institute of Tuina, Shanghai Institute of Traditional Chinese Medicine, Shanghai 200437, China. 3 Shanghai University of Traditional Chinese Medicine, Shanghai 201203, China.

First author's full name and email address: Shuaipan Zhang, 18939061729@163.com.

*Shuaipan Zhang and Zhizhen Lv contributed equally to this work.

Corresponding author and email addresses: Minfang, fangmin19650510@163.com ^#^Min Fang and Qingguang, Zhu contributed equally to this work

The rest of the authors full names and email addresses:

Zhizhen Lv, lvzhizhen1992@163.com, Qingguang Zhu, zhuqingguang@126.com; Wuquan, Sun, drsunwuquan@126.com; FeiYao, doctoryaofei@126.com; LeiFang, fanglei586@126.com; Yanbin, Cheng, cyb22011249@163.com; Zhiwei, Wu, wuzhiwei8927@163.com.

Abstract

Background: The epidemic of COVID-19 has been upgraded to a state of global pandemic around the world. Though the authorities have taken prompt medical action, more medical measures should be conducted to encounter this emergency in China. Liu-zi-jue, a traditional Chinese exercise which combines limb movements and breathing exercises, has showed that it can improve the physical and mental health of patients with lung disease. Therefore, it may have a potential efficacy of Liu-zi-jue for the patients with COVID-19. The aim of this trail is to verify whether conventional therapy plus Liu-zi-jue is superiority than conventional therapy alone for patients with COVID-19.

Methods/design: The study is a single-center randomized controlled trial with parallel-group design including two intervention groups: a conventional therapy group and a conventional therapy plus Liu-zi-jue group. A total of 186 eligible participants will be randomly assigned to the groups in a 1:1 ratio. The routine treatment of the two groups is performed daily according to guideline, and Liu-zi-jue exercise is performed twice a day until discharged. The primary outcome is the Length of Hospital Stay and Modify Borg Dyspnea Score. Secondary outcomes include Fatigue Scale-14, Patient Health Questionnaire, vital signs and respiratory symptoms. The outcomes will be assessed at three points including the baseline, before treatment on the sixth day of hospitalization and the discharge day. This study will focus on the value of a conventional therapy plus Liu-zi-jue as the treatment for COVID-19.

Discussion: This study may evaluate the efficacy of conventional therapy plus Liu-zi-jue for patients with COVID-19, which can contribute to provide a solid evidence of the complementary alternative therapy for the nationwide emergence.

Trial registration: Chinese Clinical Trial Registry, ChiCTR2000032367 Retrospectively registered on 26 April 2020.

Keywords: COVID-19, Liu-zi-jue, Conventional therapy, Randomized controlled trial

Background

Early in December 2019, an unknown acute respiratory disease now called COVID-19 appeared in Wuhan city, Hubei province, China. ^1^ A new coronavirus was isolated by high-throughput sequencing, which has been named as Severe Acute Respiratory Syndrome Coronavirus 2 (SARS-CoV-2). The World Health Organization (WHO) has defined this pneumonia as a global pandemic. ^2^Although the data show that the case fatality rate of COVID-19 is 1%, it has been reported recently that asymptomatic patients can also cause transmission, which means that it is more difficult to control. ^3, 4^ As of Apr 1, 2020, the number of confirmed diagnoses nationwide has reached 754,948, of which 36,570 were fatal cases, covering 202 countries or regions.^5^ The aged people are more susceptible to the infection and mentality has been severely affected, especially among patients and medical staff worldwide^6^. Fever and fatigue are common symptoms along with the whole process of it. ^7^ People can be infected with mild symptoms which could further develop into hypoxia, causing shock, sepsis, and even death. ^8, 9^ Patients had received oxygen therapy, antiviral, anti-infection, and other symptomatic supportive care if they were with severe symptoms. However, more effective is epidemiological screening and intensive surveillance^10^, and authorities should pay more attention to the rehabilitation of physical and mental health for mild patients with COVID-19.Exercise training is an important part of pulmonary rehabilitation and has been shown to improve dyspnea and health status and reduce medical expenses ^11^.Liu-zi-jue, a low-risk traditional Chinese exercise, which is widely used for the prevention and treatment of respiratory diseases as a complementary therapy.^12-14^ Its movements are relatively simple and are mainly composed of 6 groups of exercise combined with breathing and stretching, which is suitable for home practicing. There is no need to rely on expensive equipment and can be practiced individually or in groups without the limitation of sports experience and age. ^15^ In addition, several researches have proved that this exercise therapy are beneficial to the physical and mental health and quality of life.^16, 17^ Therefore, a randomized controlled clinical trial is being carried out to examine the efficacy of the conventional therapy plus Liu-zi-jue for the patients with COVID-19.

Methods/design

Study design

This is a single-center randomized controlled trial with two parallel arms. Recruiting patients will be carried out in Huangshi Hospital of Traditional Chinese Medicine. Data management and statistics will be conducted in the Department of Yue yang Hospital of Integrated Traditional Chinese and Western Medicine, Shanghai University of Traditional Chinese Medicine. Due to the limitations of intervention methods, only the outcome assessors and statisticians are blinded. It is planned to recruit 186 patients and randomly assign them to the conventional treatment group and the conventional treatment plus Liu-zi-jue group at a 1: 1 ratio. Liu-zi-jue exercise is conducted twice a day lasting from admission to discharge. The assessor, who was masked to the group assignment, will perform evaluation and analysis of outcome with Electronic Case Report Form (ECRF) at three points (the baseline, before treatment on the sixth day of hospitalization and the discharge day). All patients are provided with informed consent at the time of recruitment, and the trial protocol was approved by the Ethics Committee of Yue yang Hospital of Integrated Traditional Chinese and Western Medicine (item number:2020-002). The study was registered with the China Clinical Trial Registry (ChiCTR2000029978). The trial flow chart and research design are shown in Figure 1 and Figure 2 respectively.

Participants recruitment

The trial will recruit clinical patients with mild and general symptom. The participants will be diagnosed by the criteria of the National Diagnostic and Treatment Protocol for Novel Coronavirus Pneumonia (The 7th Trial Version)^18^. Participants are recruited from inpatients of the Department of Infectious Diseases of the two hospital in Hubei Province.

Inclusion criteria

(1) There is no restrictions on gender and age; (2) Patients diagnosed with mild pneumonia (slight clinical symptoms, no pneumonia manifestations on imaging), general pneumonia patients (with fever, respiratory tract symptoms, etc., imaging showed pneumonia but no multiple organ damage)^19^ (3) Hospitalized patients; (4) Volunteer to join the trial and sign the "informed consent”. (5) Promise not to perform other exercise activities.

Exclusion criteria

One of the following conditions cannot be included in this test. (1) patients with severe diseases such as cardiovascular, cerebrovascular, hematopoietic, digestive system or mental illness; (2 )pregnant and lactating women; (3) respiratory frequency> 30 times /min, showing respiratory failure; (4) complicated with other organ failure requires treatment by respiratory intensive care unit (ICU); (5) those who do not want to join the trial.

Drop out and suspension criteria

During the intervention period, patients have the right to withdraw for any reason in accordance with the Patient Management and Protection Regulations. One of the following conditions is considered the withdrawal criteria: (1) the patient did not implement the treatment plan as planned; (2) participated in other exercise programs during the trial; (3) case data are incomplete and affect the judgement of curative effect; (4) unbearable adverse events; (5) patients voluntarily withdrew on their own. It is worth noting that the trial will be immediately terminated with poor clinical efficacy or severe adverse events such as respiratory failure, severe acid-base balance disorders, sepsis and even shock.

Randomization

Clinical researchers will get a random sequence number which is automatically generated by a random number generator (IBM Corp., Armonk, NY, USA), and sequentially number them in an opaque envelope. Researchers will open random allocation envelopes and assign participants accordingly. Eligible patients will be randomly divided into a conventional treatment group and a conventional treatment plus exercise training group, with 93 patients in each group.

Blinding

Participants and Liu-zi-jue trainers are unable to be blind about group assignments due to the specific intervention, but the trainers will not know the assessment of outcomes. To reduce the risk of bias, evaluators, data managers, and statisticians were unaware of group assignments in the outcome evaluation process and data analysis. Only the data collection had been finished can the blinding procedure end.

Interventions

Both groups of participants will receive convention treatment. Based on this, the experimental group will perform Liu-zi-jue twice a day until the discharge day. The daily routine treatment plan must be recorded in the electronic medical record by the doctor's system. In order to avoid cross-infection and ensure the identity of the training content, the training method will be presented to the subjects in text and video, including exercise action essentials, time, intensity, and precautions. It should be emphasized that during the test, other exercise rehabilitation methods, including aerobic exercise, stretching exercise, yoga and other sports therapy will be prohibited. If the subject receives any other exercise regimen, the changes should be recorded on the ECRF each time.

Control group (Conventional treatment)

For patients with mild and common pneumonia, doctors strictly follow the general treatment plan " (The 7^th^ Trial Version)" for symptomatic supportive treatment. It mainly includes (1) rest in bed, strengthen supportive treatment to ensure sufficient heat; pay attention to water and electrolyte balance to maintain internal environment stability; closely monitor vital signs, oxygen saturation, etc. ; (2) monitor blood routine, urine routine, liver based on the condition function, renal function, myocardial enzymes, coagulation function, chest imaging, etc. ;(3) timely effective oxygen therapy measures, including nasal catheter, mask oxygen and trans nasal high-flow oxygen therapy. (4) antiviral therapy, although no effective antiviral therapy has been confirmed. α-interferon atomized inhalation is suggested to use (5 million U each time for adults, add 2ml of sterilized water for injection, 2 times a day) (5) antibiotic treatment will be given if a bacterial infection is confirmed diagnosis. (6) accurate Chinese medicine treatment.

Intervention group (Conventional treatment plus Liu-zi-jue)

Liu-zi-jue exercise has been operated strictly in accordance with the 2003 National General Administration of Sports standards. ^20^Its action essentials are relatively simple, suitable for everyone to master in a short time. The 6 detailed movement are as follows, which is also shown in Figure 3. (1) Put hands on the sides of the waist with palms up and stretch out your right hand towards left front 45°, and say "Xu”, then return. Complete the mirror action with the other hand as a set of actions. (2) Bend your knees and raise your arms forward with palms up. Then press your palms down, saying “He” and return. (3) Put your hands in front of your belly with palms turned inward. Stretch forward with circle shape, and say "Hu", then return. (4) Raise your hands over chest, and push hands forward with standing palms, saying “Si”, then return. (5) Put hands in front of the abdomen, wrap your hands around to the sides of your body and press down, saying "Chui”, then return. (6) Put hands in front of the abdomen, raise hands above the head and open arms. Pull back and press down, saying "Xi", then return. Each set of actions needs to be repeated three times before the next action is performed, and the patients are required to complete it all at once.

Outcome measurements

All outcome will be managed by researchers masked to the group assignment, which include three points (the baseline, before treatment on the sixth day of hospitalization and the discharge day). We mainly focus on the symptoms of dyspnea and fatigue in patients, and the degree of psychological depression is also valued. Additionally, vital signs and common symptoms of lung disease will be monitored. Primary outcome measurement.

Primary outcome measurement

LHS

The key to treatment is to shorten the hospitalization time So, we treat length of hospital stays as the primary outcome.

MBDS^21^

MBDS is a modified dyspnea questionnaire with good reliability and validity, which we list it as the main outcome index. It mainly includes 10 items about dyspnea and fatigue, and the subjects will select corresponding items according to the severity of their symptoms to obtain different scores. The "difficult breathing and fatigue that I didn't feel at all" scored 0 points. The extreme degree of breathlessness and fatigue scored 10 points. The higher the score, the more serious the condition. The MBDS will be assessed using a repeated longitudinal analysis.

Secondary outcome measurement

PHQ-9^22^

The PHQ-9 is a brief tool to assess the psychological condition of patients during illness which can lead depressive symptoms. As a self-administered questionnaire, It has been widely used to in many countries to assess the severity of depression.PHQ-9 is a self-emotion evaluation involving 9 negative things in life which is divided into 4 levels for the frequency of depression. For example, the first question: "No interest in doing something", the results of the evaluation are: "It will never happen at all, once every few days, no more than once a week, almost every day." Subjects will fill out this scale by themselves at three time points through CRFs. A repeated longitudinal analysis will also be used to evaluate the. PHQ-9.

FS-14^23^

Due to the psychological and physical damage caused by the disease, the patient will always feel tired during the illness, which is an important clinical symptom that should be monitored. Fatigue scare-14, as a widely used self-assessment scale is to measure the severity of fatigue. It has been applied in epidemiological studies to evaluate the extent of fatigue and has been proven its good effectiveness and internal reliability^24^. The 14 questions are raised by various experts in the field, which are mainly divided into two aspects: physical fatigue and mental fatigue. We will assess the FS-14 with a repeated longitudinal analysis.

Vital signs^25^.

Blood pressure, heart rate, respiration, and blood oxygen saturation will be monitored three times a day by the nurse and then an average data will be obtained.

Respiratory symptoms.

We will focus on the symptoms of patients' lungs, such as cough, expectoration, chest tightness, dyspnea, sore throat, stuffy nose, runny nose, poor diet, muscle pain, fatigue, nausea, headache. The severity of each symptom is assigned a scale of 0-10, with 0-5 being mild, greater than 5 being severe, and 10 being the most severe. Each participant will score their own symptoms.

Safety evaluation

The doctors should focus on the vital signs of patients with COVID-19strictly to decrease the incidence of adverse events, which is refer to the unexpected responses that occur during or after treatment. Record and analyze them whether the adverse events are related to the treatment. Liver function and renal function tests and lung CT imaging tests also need to be monitored.

Data collecting and monitoring

During the recruitment phase, screeners will collect demographics of the participants. The data assessor will record the baseline characteristics of the patient and the outcomes through a CRFs. Next, two third-party personnel who have received strict data management training will receive data in the form of an excel database, and then they will enter the real-time data into the China Clinical Trial Registration Center. This electronic data management system will be used in the Ministry of Science and Technology of Yue yang Hospital of Integrated Traditional Chinese and Western Medicine to collect and monitor test data in real time.

Statistical analyses

Statisticians will use SPSS Windows version 21.0 software to perform the data analysis within group and between groups. Data will be expressed as mean ± standard deviation. The data between groups will be compared by Student’s t test when the continuous variables can meet a normal distribution or T distribution. Otherwise, Mann–Whitney test or Wilcoxon test will be used. For categorical data, the Fisher’s exact or the Chi-square test will be adopted. We will conduct an intention-to-treat analysis if participants are lost to follow-up and perform a simple correlation analysis or simple regression analysis to determine the potential correlation between the outcome measurements. Adverse events in each group will be documented as percentage (%) for safety assessments using the chi-square test or Fisher’s exact test. All statistical analyses will be conducted in a two-sided manner, with a significance level of 5%.

Sample size calculation

The trial takes the LHS as the primary efficacy outcome. With reference to the latest literature^26^, it is assumed that the average length of stay in the control group of this study is 10 days, the standard deviation is 3 days, and the average length of stay in the treatment group is 8.5 days, α = 0.05 , β = 0.10. Considering the 10% drooping rate, it was calculated that 93 subjects were needed in each group, and a total of 186 cases were required to be recruited.

Quality control

Quality control will be conducted during the processing of the trail under the management of the steering committee. Professional trial methodology should be trained before the researchers participate in the trial which can ensure the consistency of methods. Anything modification in the study protocol happen will inform the steering committee and ethics committee

Discussion

The epidemic of COVID-19 has become a worldwide public health problem^27^. Although prompt emergency measures have been taken, the number of people with the disease has been increasing since the onset of the disease in China. Data from a clinical epidemic model showed that the reproduction number (R_0_) has reached 2.68^28^. The current effective measures are to strictly epidemic prevention and control measures. The aged with a weak immune system show a worse prognosis and the sudden medical burden has caused a great psychological problem on patients and the medical workers^29^. Only symptomatic supportive treatment can be used to reduce complications and prevent disease progression due to the shortage of antiviral drugs. Dyspnea and fatigue are the symptoms that best reflect the severity of a patient's illness. TCM is a discipline that attaches great importance to symptoms. Liu-zi-jue, as part of TCM, has been proven in many ways to significantly improve lung function such as dyspnea and fatigue and mental health, which may play an antiviral role at early stage^30-33^. The Yellow Emperor's Canon of Internal Medicine states that " a body full with healthy Qi energy will let the evil Qi energy go away “. It can increase the body's ability to resist external evil through activities. The breathing method improve chest movement and lung ventilation as well as improve overall health by improving diaphragm muscle movement, which is the most important breathing muscles in the body^34^. It is suitable for frail patients and seniors in the hospital status with the characterization of slow motion and respiration and relaxation. By training in body posture and movement, regulating breathing patterns and maintaining mental calmness, a variety of natural self-regulation and self-repair mechanisms in body can be activate to stimulate the balanced release of endogenous neurohormones^35^. However, further clinical evidence is needed for the specific clinical effectiveness of the Liiu-zi-jue exercise therapy. This protocol is suit to the research methodology of evidence-based medicine to ensure the reliability of clinical trials maximally. It is used to verify the clinical effectiveness of Liu-zi-jue for the recovery of patients with COVID-19.

Study limitations

There is an inevitable limitation that it is difficult to control the methodology of blinding during the physical intervention. In this study, Liu-zi-jue as a physical therapy is impossible to be blind for the participants and therapist. We have not conducted a comparison of efficacy between two different exercises.

Trial status

This trial is recruiting patients now. This trial was registered retrospectively in the Chinese Clinical Trial Registry on 26 April 2020. The registration number is ChiCTR2000032367.The protocol is Version 2, March 4.

Abbreviations

2019 Novel Coronavirus Pneumonia: COVID-19; CRFs: Clinical Report Forms; FS-14: Fatigue Scale-14; HR: Heart Rate; LHS: Length of Hospital Stay; MBDS: Modify Borg Dyspnea Score PHQ-9: Patient Health Questionnaire; WHO: World Health Organization.

Acknowledgements

Not applicable

Availability of data and material

Not applicable.

Funding

This study is supported financially by the project of Emergency scientific research project for prevention and control of new coronavirus (COVID-19) by Shanghai University of Traditional Chinese Medicine (first batch)（No fund number）and Shanghai's three-year action project on further accelerating the development of Traditional Chinese Medicine (ZY (2018-2020)-CCCX-2004-02). The funding is widely used and applicable to this study. The funder had no role in the design of the study, analysis, collection, and interpretation of the data, or the writing and decision for publication of the manuscript.

Authors’ contributions

SZ planned the study protocol and drafted the manuscript. ZL planned the study protocol and participated in the critical revision of the manuscript. QZ, carried out the Liu-zi-jue movements for patients and participated in the design of the protocol. MF managed the study and reviewed the manuscript. WS, FY, LF participated in designing the trial and helped to prepare the manuscript. ZW participated in the remote intervention supervision and revised the manuscript. All the authors have read and approved the final manuscript.

Ethics approval and consent to participate

Ethics approval was requested and granted by the ethics committee of Yue yang Hospital of Integrated Traditional Chinese and Western Medicine (item number :2020-002). Informed consent will be obtained from all study participants before starting any data collection by the clinical trial communicator. All participants will provide their consent in writing. Nobody except the investigators have access to the final data.

Consent for publication

Not applicable

Competing interests

The authors declare that they have no competing interests.

References:

**1.** Huang C, Wang Y, Li X, et al. Clinical features of patients infected with 2019 novel coronavirus in Wuhan, China. *Lancet (London, England).* 2020;395(10223):497-506.

**2.** Carlson CJ. From PREDICT to prevention, one pandemic later. *The Lancet Microbe.* 2020.

**3.** Gates B. Responding to Covid-19 — A Once-in-a-Century Pandemic? *New Engl J Med.* 2020.

**4.** Hoehl S, Rabenau H, Berger A, et al. Evidence of SARS-CoV-2 Infection in Returning Travelers from Wuhan, China. *New Engl J Med.* 2020.

**5.**  World Health Organization home page.(https://experience.arcgis.com/experience/685d0ace521648f8a5beeeee1b9125cd)(Apr 1，2020)，Assessed 1th Apr 2020.

**6.** W C, G G, BD K. Mental health in the Covid-19 pandemic. *QJM : monthly journal of the Association of Physicians.* 2020.

**7.** Holshue ML, DeBolt C, Lindquist S, et al. First Case of 2019 Novel Coronavirus in the United States. *New Engl J Med.* 2020.

**8.** Jin Y, Cai L, Cheng Z, et al. A rapid advice guideline for the diagnosis and treatment of 2019 novel coronavirus (2019-nCoV) infected pneumonia (standard version). *Military Medical Research.* 2020;7(1):4.

**9.** Minodier L, Charrel RN, Ceccaldi P, et al. Prevalence of gastrointestinal symptoms in patients with influenza, clinical significance, and pathophysiology of human influenza viruses in faecal samples: what do we know? *Virol J.* 2015;12:215.

**10.** Chen S, Yang J, Yang W, Wang C, Bärnighausen T. COVID-19 control in China during mass population movements at New Year. *The Lancet.* 2020.

**11.** Divo M, Pinto-Plata V. Role of exercise in testing and in therapy of COPD. *The Medical clinics of North America.* 2012;96(4):753-766.

**12.** Guo Y, Xu M, Ji M, et al. Effect of Liuzijue Qigong on patients with chronic obstructive pulmonary disease: Protocol for a systematic review and meta-analysis. *Medicine.* 2018;97(40):e12659.

**13.** Wu W, Liu X, Li P, Li N, Wang Z. Effect of Liuzijue Exercise Combined with Elastic Band Resistance Exercise on Patients with COPD: A Randomized Controlled Trial. *Evidence-based complementary and alternative medicine : eCAM.* 2018;2018:2361962.

**14.** Gendron LM, Nyberg A, Saey D, Maltais F, Lacasse Y. Active mind-body movement therapies as an adjunct to or in comparison with pulmonary rehabilitation for people with chronic obstructive pulmonary disease. *The Cochrane database of systematic reviews.* 2018;10(10):D12290.

**15.** Yeh GY, Wang C, Wayne PM, Phillips R. Tai chi exercise for patients with cardiovascular conditions and risk factors: A SYSTEMATIC REVIEW. *J Cardiopulm Rehabil Prev.* 2009;29(3):152-160.

**16.** Wang X, Pi Y, Chen B, et al. Effect of traditional Chinese exercise on the quality of life and depression for chronic diseases: a meta-analysis of randomised trials. *Sci Rep-Uk.* 2015;5:15913.

**17.** Li Z, Liu S, Wang L, Smith L. Mind-Body Exercise for Anxiety and Depression in COPD Patients: A Systematic Review and Meta-Analysis. *Int J Env Res Pub He.* 2019;17(1):22.

**18.** National Health Commission of the People ’s Republic of China home page.(Http://www.nhc.gov.cn) ,(Mar 4, 2020) (Assessed on Mar 22th 2020).

**19.** 新型冠状病毒感染的肺炎诊疗方案(试行第五版). *中国中西医结合杂志.*:1-3.

**20.** Wang C, Yu L, Yang J, Wang RW, Zheng YN, Zhang Y. Effectiveness of LiuZiJue Qigong versus traditional core stability training for poststroke patients complicated with abnormal trunk postural control: study protocol for a single-center randomized controlled trial. *Trials.* 2020;21(1):254.

**21.** Ijiri N, Kanazawa H, Yoshikawa T, Hirata K. Application of a new parameter in the 6-minute walk test for manifold analysis of exercise capacity in patients with COPD. *Int J Chronic Obstr.* 2014;9:1235-1240.

**22.** Urtasun M, Daray FM, Teti GL, et al. Validation and calibration of the patient health questionnaire (PHQ-9) in Argentina. *Bmc Psychiatry.* 2019;19(1):291.

**23.** Jing M, Lin W, Wang Q, et al. Reliability and Construct Validity of Two Versions of Chalder Fatigue Scale among the General Population in Mainland China. *Int J Env Res Pub He.* 2016;13(1):147.

**24.** Chalder T, Berelowitz G, Pawlikowska T, et al. Development of a fatigue scale. *J Psychosom Res.* 1993;37(2):147-153.

**25.** Mok WQ, Wang W, Liaw SY. Vital signs monitoring to detect patient deterioration: An integrative literature review. *Int J Nurs Pract.* 2015;21 Suppl 2:91-98.

**26.** Guan WZNN. Clinical characteristics of 2019 novel coronavirus infection in China. *medRxiv.* 2020.

**27.** Wang C, Horby PW, Hayden FG, Gao GF. A novel coronavirus outbreak of global health concern. *Lancet.* 2020;395(10223):470-473.

**28.** Huang C, Wang Y, Li X, et al. Clinical features of patients infected with 2019 novel coronavirus in Wuhan, China. *Lancet (London, England).* 2020:S140-S6736.

**29.** Kang L, Li Y, Hu S, et al. The mental health of medical workers in Wuhan, China dealing with the 2019 novel coronavirus. *The lancet. Psychiatry.* 2020:S2215-S2366.

**30.** Campo RA, Agarwal N, LaStayo PC, et al. Levels of fatigue and distress in senior prostate cancer survivors enrolled in a 12-week randomized controlled trial of Qigong. *Journal of cancer survivorship : research and practice.* 2014;8(1):60-69.

**31.** Craske NJM, Turner W, Zammit-Maempe J, Lee MS. Qigong ameliorates symptoms of chronic fatigue: a pilot uncontrolled study. *Evidence-based complementary and alternative medicine : eCAM.* 2009;6(2):265-270.

**32.** Tong H, Liu Y, Zhu Y, Zhang B, Hu J. The therapeutic effects of qigong in patients with chronic obstructive pulmonary disease in the stable stage: a meta-analysis. *Bmc Complem Altern M.* 2019;19(1):239.

**33.** Zhang H, Li J, Yu X, et al. An evaluation of activity tolerance, patient-reported outcomes and satisfaction with the effectiveness of pulmonary daoyin on patients with chronic obstructive pulmonary disease. *Int J Chronic Obstr.* 2017;12:2333-2342.

**34.** 胡尧, 邬建卫, 阳仁均. 传统健身气功五禽戏与太极拳对心肺功能影响的对比研究. *当代体育科技.* 2014;4(28):10-12.

**35.**

The People's Government of Hubei Province

The announcement from the Hubei Provincial Novel Coronavirus Pneumonia Prevention and Control Headquarter

http://www.hubei.gov.cn/zhuanti/2020/gzxxgzbd/zxtb/202002/ (Feb 20, 2020), Accessed 29th Feb 2020

([in Chinese]).
