# Supplementary material for: Efficacy of Liu-zi-jue in Patients with 2019 Novel Coronavirus Pneumonia (COVID-19): structured summary of a study protocol for a randomized controlled trial
Source: Trials. 2020 May 23;21:416. doi: 10.1186/s13063-020-04383-2 (PMC7245505; doi:10.1186/s13063-020-04383-2)
Supplement: Supplementary file 3 — Additional file 3. Proof of Ethics. [file 13063_2020_4383_MOESM3_ESM.pdf]

8. 研究团队（组长单位）中建议增加呼吸内科相关专业的研究者。

按审查意见修改后的文件，或对审查意见不同观点的陈诉，请提交“复审申请”，方案 / 知情同意书请注明新的版本号和版本日期，并以阴影和 / 或下划线方式标注修改部分，报伦理委员会审查，经批准后执行。

| | |
|---|---|
| 调整的年度/定期跟踪审查频率 | NA |
| 伦理委员会 | 上海中医药大学附属岳阳中西医结合医院伦理委员会 |
| 联系人与联系电话 | 伦理委员会秘书：殷从[illegible]；联系电话：021-65161782-8122 |
| 主任委员签名 | [illegible] |
| 日期 | 2020 年 2 月 18 日 |

# 伦理审查意见

| | |
|---|---|
| 意见号 | 2020-002-01 |
| 项目名称 | 中医传统功法防控新冠肺炎技术应用研究 |
| 申办者 | 上海中医药大学附属岳阳中西医结合医院：房敏 |
| 研究单位 | 上海中医药大学附属岳阳中西医结合医院、黄石市中医医院、湖北省中医院 |
| 主要研究者 | 房敏 |
| 审查类别 | 初始审查 |
| 审查方式 | 远程视频会议 |
| 审查日期 | 2020 年 2 月 18 日 |
| 审查委员 | 郑　莉、张春雁、樊民胜、姚永其、徐玲玲、马晓芃、黄　瑾、史　晓、孙武权、冯寿全、张婷婷 |
| 审查文件 | 1. 初始伦理审查申请<br>2. 研究者经济利益声明<br>3. 临床研究方案（版本号：V1.0；版本日期：2020.2.15）<br>4. 知情同意书（版本号：V1.0；版本日期：2020.2.15）<br>5. 招募广告（版本号：V1.0；版本日期：2020.2.15）<br>6. 病例报告表（版本号：V1.0；版本日期：2020.2.15）<br>7. 受试者身份识别代码表<br>8. 主要研究者简历、培训证书<br>9. 项目任务书 |

**审查意见**

根据卫生部《涉及人的生物医学研究伦理审查办法》（2016）、NMPA《药物临床试验质量管理规范》、《医疗器械临床试验管理规范（2016）》、WMA《赫尔辛基宣言》和 CIOMS《人体生物医学研究国际道德指南》的伦理原则，经本伦理委员会审查，意见如下：

1. 排除标准中建议再补充排除合并糖尿病、肿瘤等不适合参加本研究的其他疾病患者。
2. 请注意实时更新研究方案中的《新型冠状病毒感染的肺炎诊疗方案》。
3. 对于重症转为轻症的患者是否可以纳入本研究，请在方案中明确。
4. 由于本研究是异地开展，请在研究方案中补充介绍如何保证 2 个分中心研究者的功法培训到位，如何确保干预措施实施到位？
5. 知情同意书第 3 页“3.需要您配合的其他事项”部分第 2 段内容“在研究期间您不能进行其他的运动锻炼或参加其他治疗新冠的项目”表述不妥当，常规治疗中可能会有这些治疗手段，建议修改表述。
6. 知情同意书第 3 页“5.参加研究可能的不良反应、风险和不适、不方便”部分请补充介绍由于戴口罩进行功法锻炼引起的不适，以及如果因戴口罩进行功法锻炼引起不适进而中止锻炼后如何处理，同时补充介绍如果疾病进展严重后如何处理。
7. 补充介绍如何和患者知情同意书，请注意避免传染疾病，建议可以以视频的形式进行知情同意书并留取过程录像。

# 伦理审查批件

| | |
|---|---|
| 批件号 | 2020—002 |
| 项目名称 | 中医传统功法防控新冠肺炎技术应用研究 |
| 申办者 | 上海中医药大学附属岳阳中西医结合医院：房敏 |
| 研究单位 | 上海中医药大学附属岳阳中西医结合医院、黄石市中医医院、湖北省中医院 |
| 主要研究者 | 房敏 |
| 审查类别 | 初始审查，初始审查后的复审（重审） |
| 审查方式 | 远程会议审查 |
| 审查日期 | 2020 年 2 月 18 日，2020 年 2 月 19 日 |
| 审查委员/秘书 | 郑　莉、张春雁、樊民胜、姚永其、徐玲玲、马晓芃、黄　瑾、史　晓、冯寿全、王雪文 |
| 批准文件 | 1. 初始伦理审查申请<br>2. 研究者经济利益声明<br>3. 临床研究方案（版本号：V1.1；版本日期：2020.2.18）<br>4. 知情同意书（版本号：V1.1；版本日期：2020.2.18）<br>5. 招募广告（版本号：V1.1；版本日期：2020.2.18）<br>6. 病例报告表（版本号：V1.1；版本日期：2020.2.18）<br>7. 受试者身份识别代码表<br>8. 主要研究者简历、培训证书<br>9. 项目任务书 |

**审查意见**

根据卫生部《涉及人的生物医学研究伦理审查办法》（2016）、NMPA《药物临床试验质量管理规范》、《医疗器械临床试验管理规范（2016）》、WMA《赫尔辛基宣言》和 CIOMS《人体生物医学研究国际道德指南》的伦理原则，经本伦理委员会审查，同意按所批准的临床研究方案、知情同意书、招募材料开展本项研究。

请遵循 GCP 原则、遵循伦理委员会批准的方案开展临床研究，保护受试者的健康与权利。

研究开始前，请申请人完成临床试验注册。

研究过程中若变更主要研究者，对临床研究方案、知情同意书、招募材料等的任何修改，请申请人提交修正案审查申请。

发生严重不良事件，请申请人及时提交严重不良事件报告。

请按照伦理委员会规定的年度 / 定期跟踪审查频率，申请人在截止日期前 1 个月提交研究进展报告；申办者应当向组长单位伦理委员会提交各中心研究进展的汇总报告；当出现任何可能显著影响试验进行、或增加受试者危险的情况时，请申请人及时向伦理委员会提交书面报告。

研究纳入了不符合纳入标准或符合排除标准的受试者，符合中止试验规定而未让受试者退出研究，给予错误治疗或剂量，给予方案禁止的合并用药等没有遵从方案开展研究的情况；或可能对受试者的权益 / 健康以及研究的科学性造成不良影响等违背 GCP 原则的情况，请申办者 / 监察员 / 研究者提交违背方案报告。

申请人暂停或提前终止临床研究，请及时提交暂停 / 终止研究报告。

完成临床研究，请申请人提交研究完成报告。

| | |
|---|---|
| 年度/定期跟踪审查频率 | ■ 3 个月，口 6 个月，12 个月 |
| 批件有效期 | 2020 年 2 月 19 日——2020 年 2 月 18 日 |
| 联系人与联系电话 | 伦理委员会秘书：殷[illegible]合　联系电话：021-65161782-8122 |
| 主任委员签名 | [illegible] |
| 伦理委员会 | 上海中医药大学附属岳阳中西医结合医院伦理委员会（盖章） |
| 日期 | 2020 年 2 月 19 日 |

| | |
|---|---|
| Approval number | 2020-002 |
| Project name | Efficacy of Liu-zi-jue in patients with 2019 novel coronavirus pneumonia: study for a randomized controlled trial |
| Applicant | Fang Min |
| Research unit | Yueyang Hospital of Integrated Traditional Chinese<br>and Western Medicine affiliated to Shanghai University of Traditional Chinese Medicine |
| Researcher leader | Fang Min |
| Review category | Re-examination after initial review |
| Mode of review | Remote conference review |
| Review date | February 19, 2020 |
| Review committee, secretary | Zheng Li, Zhang Chunyan |
| Approval documents | 1. Initial ethical review application<br>2. Study economic benefits<br>3. Clinical research program<br>4. Informed consent<br>5. Recruitment advertising<br>6. Case report form<br>7. Key researcher's resume and training certificate<br>8. Project task book |
| Review opinion | |

房敏

According to the Ministry of Health's "Ethics Review of Human Biomedical Research"
(2016), CFDA "Quality Management Standards for Clinical Trials", "Medical Device Clinical
Trial Management Regulations (2016)", WMA "Helsinki Declaration" and CIOMS "Human
Biology The ethical principles of the International Code of Ethics for Medical Research
were reviewed by the Ethics Committee and agreed to conduct this study in accordance
with approved clinical research protocols, informed consent, and recruitment materials.
Follow the GCP principles and follow a protocol approved by the Ethics Committee to
conduct clinical research to protect the health and rights of the subjects.
Before the study begins, the applicant is requested to complete the clinical trial registration.
If the main investigator is changed during the research process, any changes to the
clinical research plan, informed consent, recruitment materials, etc., the applicant is
requested to submit an amendment review application.
Serious adverse events occurred, and applicants are required to submit reports of serious adverse events in a timely manner.
Please follow the frequency of the annual follow-up review as stipulated by the ethics
committee. The applicant submits the research progress report one month before the
deadline. If there is a violation of the program during the trial, it is necessary to submit

| | |
|---|---|
| Annual review frequency | 3 month |
| Validity period | February 19, 2020 to February 19, 2021 |
| Contact and contact number | secretary of the Ethics Committee Congquan Yin ,telephone number 021-65161782-8122 |
| Signature of the chairman | Zheng Li |
| Ethics committee | Ethics committee of Yueyang Hospital of Integrated Traditional Chinese and Western Medicine affiliated to Shanghai University of Traditional Chinese Medicine |
| Date | 19-Feb-20 |
