# Supplementary material for: Efficacy of Liu-zi-jue in Patients with 2019 Novel Coronavirus Pneumonia (COVID-19): structured summary of a study protocol for a randomized controlled trial
Source: Trials. 2020 May 23;21:416. doi: 10.1186/s13063-020-04383-2 (PMC7245505; doi:10.1186/s13063-020-04383-2)
Supplement: Supplementary file 4 — Additional file 4. Funding proof. [file 13063_2020_4383_MOESM4_ESM.pdf]

上海中医药大学防治新型冠状病毒（2019-nCoV）

应急科研攻关项目

申　请　书

项目名称： 中医传统功法防控新冠肺炎技术应用研究

申 请 者： 房 敏

所在单位： 上海中医药大学针推学院

研究期限： 2020 年 2 月-2020 年 7 月

上海中医药大学科技处

2020 年 2 月

Shanghai University of Traditional Chinese Medicine

Prevents and Controls New Coronavirus (2019-nCoV)

Emergency scientific research project

Application

Project Title: Application of Traditional Chinese Exercise-Dao Yin in Prevention and Control of novel coronavirus pneumonia

Applicant: Fang Min 方敏

Affiliation: Yue yang Integrated Hospital of Traditional Chinese and Western Medicine, Shanghai University of Traditional Chinese Medicine

Study period: February 2020-July 2020

Department of Science and Technology, Shanghai University of Traditional Chinese Medicine

February 2020

附件 1

上海市进一步加快中医药事业发展三年行动计划（2018 年-2020 年）

项 目 建 设 任 务 书

项目编号　ZY(2018-2020)-CCCX-2004-02

项目类别　中医药传承创新平台建设

项目名称　推拿生物学效应机制与应用创新研究

项目负责人　房敏

项目承担单位　上海中医药大学附属岳阳中西医综合医院（盖章）

项目起止年月　2018 年 9 月　至　2020 年 12 月

上海市卫生和计划生育委员会

上海市中医药发展办公室

二〇一八年制

Shanghai Further accelerate the three-year action plan for the development of Chinese medicine program planning proposal

Project Number:ZY(2018-2020)-CCCX-2004-02

Project type: Construction of Chinese Medicine Heritage and Innovation Platform

Project name: The research of Biological effect mechanism , application and innovation with Tuina intervention

Project manager: Min Fang 房敏

Project support unit: Yueyang Hospital of Integrated Traditional Chinese and Western Medicine affiliated to Shanghai University of Traditional Chinese Medicine

Start and end dates: September 1, 2018 to December 31, 2020

Shanghai Health and Planning Commission Shanghai

Chinese Medicine Development Office

Produced in 2018
